# Chronic toxicity studies of *gandhaka rasayana* - A herbo-mineral preparation used in Ayurvedic practice

**DOI:** 10.1016/j.jaim.2021.05.011

**Published:** 2021-11-01

**Authors:** Ravi Mundugaru, Shrinidhi R. Ballal, Sudhakar Bhat, Ravishankar Basavaiah

**Affiliations:** a Department of Pharmacology & Toxicology, SDM Centre for Research in Ayurveda and Allied Sciences, Udupi, 574118, Karnataka, India; b Department of Agadatantra, Sri Dharmasthala Manjunatheshwara College of Ayurveda, Kuthpady, Udupi, 574118, India

**Keywords:** Chronic toxicity, *Gandhaka rasayana*, Histopathology

## Abstract

**Background:**

*Gandhaka rasayana* (GR) is an important component of many Ayurvedic formulations besides being used as a standalone therapy. However, literature review revealed a chronic toxicity studies with longer duration.

**Objectives:**

To delineate the safety profile of GR for 180 days administration in rats.

**Materials and methods:**

Wistar albino rats of both sexes weighing 150 ± 10 g body weight in groups of 20 (10 male and 10 female) for each of the three GR dose levels i.e., 0.54 g, 1.08 g, and 5.4 g/kg were employed. Carboxyl methyl cellulose was administered to the control group in equal volume. Toxicity was assessed based on the changes observed, compared to control, in body weight gain, food and water consumption, organ weight and histopathology, clinical biochemistry, and hematological parameters as per AYUSH guidelines.

**Results:**

GR repeated dose administration caused significant changes in body weight gain, organ ponderal changes, few hematologic and biochemical parameters. Male rats administered with GR at 1.08 g/kg dose showed a significant decrease in the MCV and MCH compared to control, whereas female rat’s administred with 1.08 g/kg and 0.54 g/kg dose showed a significant increase in the MCV and MCH. GR administered at 0.54 g/kg showed a significant increase in the serum glucose level in male rats, whereas female rats showed a significant elevation in the cholesterol level. GR at 0.54 g/kg and 5.4 g/kg showed a significant elevation in the serum SGPT level in male rats. These changes were not observed in female rats. Histological examination revealed mild pathological changes in organs like kidney, liver, spleen and jejunum.

**Conclusion:**

The data generated shows that GR is safe and does not have any toxicity potential at the doses used in therapeutics. Some of the changes observed were at higher dose levels which is not likely to be used clinically.

## 1 Introduction

There are many metal and mineral-based formulations used in Ayurveda and other Indian systems of medicines with widespread biological activities. Scientific data on the safety aspects of these formulations can increase their acceptability, especially, among the educated middle class in the country making this an important segment of drugs available to large population. They can prove to be of immense help in the treatment of chronic degenerative disorders and in areas where there is a therapeutic gap. Data generated on such under-evaluated Ayurvedic medicines will help in the global acceptance of mineral and herbo-mineral formulations [1].

*Gandhaka Rasayana* (GR) is an important herbo-mineral drug used in Ayurveda as a *Rasayana* (rejuvenator) drug. It is extensively used in a wide range of clinical conditions such as skin diseases, as an appetizer, in respiratory disorders, arthritis, allergies, pain symptoms, bladder disorder, diabetes, and cough. *Gandhaka* (sulphur) is used extensively for processing mercury for the preparation of mercury-based formulations in *Rasasastra*. The formulations derived from *Gandhaka* are known as *Gandhaka kalpas* which are extensively used in different kinds of formulations with a variety of therapeutic applications. GR is believed to favorably influence tissue repairing and rejuvenation. Sulphur containing preparations have been found to have unique activities like efficacy against sickle-cell anaemia, iron over-loading as occurs in thalassemia [2], anti-leukemic activity [3], anti-hyperlipidemic activity, and antidiabetic activity with *Trigunamakardhwaj* [4]. *Siddhmakardhwaj* containing formulation and *Swarana bhasma* are reported to possess nootrophic effect [5] while *Brihatvata* *Chinthamani Rasa*, a compound mineral preparation, widely used in the management of neuro-psychiatric illnesses has been reported to be effective in experimental stroke in rats. *Mahamrutyunjaya Rasa* another formulation is often used to treat cardiac disorders [6]. Thus, *Gandhaka-*based preparations hold an important place in Ayurvedic pharmaceutics. However, though GR has widespread clinical use, there is a lack of structured data related to its safety. Hence, the present study was designed to evaluate the chronic toxicity profile of GR.

## 2 Materials and methods

### 2.1 Procurement and preparation of test drug

The ingredients required for the preparation of GR were procured from SDM Ayurveda Pharmacy, Udupi. After proper authentication of the ingredients, GR was prepared as per the reference of *Rasaratna Samuchchaya* following Ayurveda Formulary of India (AFI) [7] at SDM Ayurveda Pharmacy, Udupi. The processing of GR involved the following steps:

1) The purified form of sulphur was finely powdered and *Bhavana* (trituration) was given with the decoction of *Chaturjata* (group of four plant ingredients such as *Ela* (*Eletteria cardomomum* Maton), *Twak* (*Cinnamonium cassia* Nees ex Blume), *Patra* (*Cinnamonium tamala* (Buch.-Ham.) T. Nees & Eberm), and *Nagakeshar* (*Mesuaferra* L), *Triphala* (*Harithaki* (*Terminalia chembula* Gaertn. Retz)), *Vibhitaki* (*Terminalia belerica* Gaertn. Roxb.) and *Amalaki* (*Embilica* *officinalis* Gaertn.), Ginger (*Zingiber officinalis* (L.) H. Karst.), and *Swarasa* (fresh juice) of *Guduchi* (*Tinospora cordifolia*), *Bhringraja* (*Eclipta alba* (L.) Hassk.), and *Adraka* (*Z. officinalis* (L.) H. Karst.) respectively for eight times each.
2) Equal parts of sugar powder was added and mixed with dried *Gandhaka*.
3) The powdered form of GR was used for its toxicity evaluation in Wistar albino rats [8,9].

### 2.2 Dose selection and administration

AFI mentions the human dose of GR as 1–3 g; however, there are a few physicians who use higher dose because of higher sugar content in the formulation. Hence, we considered a maximum tolerated dose to determine its safety in the experimental study [9,10]. The dose for experimental animals was calculated by extrapolating human dose into rat dose as 1.08 g/kg based on the body surface area ratio by referring to the standard table of Paget and Barnes [11].

### 2.3 Experimental animals

Wistar albino rats of male and female sex, weighing 150 ± 10 g were procured from animal house facility attached to the Pharmacology and Toxicology Laboratory of SDM Centre for Research in Ayurveda and Allied Sciences, Udupi. The animals were maintained at a temperature in the range of 25–27 °C, humidity of 53 ± 2%, and natural light and dark cycles. Animals were fed with commercial rat pellet diet (Sai Durga Feeds, Bengaluru) and water *ad libitum.* Prior to the experimentation, approval was obtained from Institutional Animal Ethics Committee (Reference no. SDMCRA/IAEC/SDM-04, 25/08/2015).

### 2.4 Chronic toxicity study

Chronic toxicity study was conducted as per AYUSH guidelines enunciated in Rule 170 of Drugs and Cosmetic Act [12,13]. For each dose level of GR, Wistar albino rats of both sex weighing 150 ± 10 g body weight in groups of 20 (10 male and 10 female) were used for the experimental study. Control group of rats was given 0.5% of carboxyl methyl cellulose (CMC) daily and considered as the vehicle control. Initially, the animals were distributed in different cages based on age, weight range, sex and then allotted to different experimental groups to ensure uniformity in distribution of different attributes. Chronic toxicity was assessed after single daily administration of GR at therapeutic dose 1.08 g/kg (TED), five times of therapeutic dose 5.4 g/kg (5X TED), and half the therapeutic dose 0.54 g/kg (half the TED) for a period of 180 days as solution in 0.5% CMC. Toxicity was assessed in terms of changes related to body weight, food and water consumption, organs weight and histological study of vital organs such as brain, pituitary, thymus, lymph node, heart, lung, trachea, liver, stomach strip, jejunum, spleen, kidney, testis, seminal vesicle, prostate, uterus, ovary, skin, sciatic nerve, and bone marrow [14]. Changes in biochemical parameters such as SGOT, SGPT, serum alkaline phosphatase, serum total protein, serum albumin, biliruibin, serum creatinine, serum cholesterol, serum triglyceride, blood urea, blood glucose and hematological changes such as hemoglobin, white blood cells (WBC), red blood cells (RBC), packed cell volume (PCV), mean corpuscular volume (MCV), mean corpuscular hemoglobin (MCH), red cell distribution width coefficient of variation (RD WCV), red cell distribution width standard deviation (RD WSD), mean corpuscular hemoglobin concentration (MCHC) and platelet count were estimated [15,16]. While assessing the behavioural-related changes, the evaluators were blind to the group-related information.

### 2.5 Statistical analysis

Data were expressed as mean ± SEM and analyzed using one way ANOVA followed by Dunnett's multiple comparison post hoc tests using GraphPad Prism (version 3) Software, Inc., USA. A ‘*p*’ value less than 0.05 was considered as statistically significant.

## 3 Results

### 3.1 Effect of GR on percentage body weight

Gradual time-dependent increase in body weight was observed in all the groups including control and drug administered groups. GR caused time-dependant increase in the body weight at all the three dose levels measured during the 1^st^, 4^th^, 8^th^, 12^th^, 16^th^, 20^th^ and 24^th^ weeks of the observation period and statistically significant increase was found in all the groups as compared to the control rats (p < 0.05, p < 0.01) (Table 1). The percentage change in the body weight was observed in both, the control and GR administered groups. The repeated administration of three different dose levels of GR caused time-dependant increase in the percentage change in the body weight measured during 1^st^, 4^th^, 8^th^, 12^th^, 16^th^, 20^th^ and 24^th^ weeks of observation period and the observed increase was found to be statistically significant as compared to the control rats (∗p < 0.05, ∗∗p < 0.01).

**Table 1:** Effect of GR on percentage changes in the body weight of Wistar albino rats.

Group	Control		GR (TED X ½)		GR (TED)		GR (5X TED)	
	Male	Female	Male	Female	Male	Female	Male	Female
1st week	6.19 ± 0.93	7.37 ± 1.47	20.68 ± 1.45∗∗	10.51 ± 3.20	21.97 ± 1.98∗∗	15.65 ± 1.41∗	36.84 ± 3.18∗∗	28.05 ± 1.22∗∗
4th week	33.68 ± 2.46	26.48 ± 4.63	51.49 ± 4.44	39.75 ± 3.49	71.84 ± 3.22∗∗	49.53 ± 4.35∗	116.54 ± 9.14∗∗	79.14 ± 8.31∗∗
8th week	44.54 ± 4.59	26.70 ± 4.33	93.83 ± 6.24∗∗	71.17 ± 6.37∗∗	137.95 ± 6.63∗∗	88.32 ± 6.87∗∗	207.49 ± 15.6∗∗	132.05 ± 12.1∗∗
12th week	57.37 ± 2.92	44.99 ± 6.55	141.78 ± 5.76∗∗	91.00 ± 9.10∗	182.70 ± 4.87∗∗	110.89 ± 6.28∗∗	274.93 ± 15.49∗∗	157.51 ± 16.87∗∗
16th week	61.61 ± 2.90	48.37 ± 6.00	170.34 ± 10.92∗∗	102.78 ± 9.33∗	218.28 ± 3.75∗∗	119.56 ± 9.79∗∗	314.15 ± 18.20∗∗	176.91 ± 26.13∗∗
20th week	92.84 ± 1.94	68.78 ± 4.03	192.11 ± 10.54∗∗	115.98 ± 6.61∗	254.59 ± 5.03∗∗	130.45 ± 10.87∗∗	330.40 ± 20.23∗∗	184.92 ± 25.08∗∗
24th week	119.45 ± 2.16	94.44 ± 2.46	203.97 ± 10.38∗∗	118.77 ± 6.89	264.99 ± 7.49∗∗	133.31 ± 11.17	369.90 ± 21.91∗∗	203.97 ± 27.65∗∗

Data: Mean ± SEM, p < 0.05, p < 0.01 in comparison to control group. GR- *Gandhaka rasayana*.

### 3.2 Effect of GR on organs weight

The test drug had no effect on weight of organs such as brain, heart, spleen, trachea, testis, ventral prostrate, and uterus as compared to the control group. The test drug administered at three different dose levels showed significant reduction in the lung, liver weight and significant increase in the stomach and seminal vesicle weight as compared to normal control rats (Table 2). The test drug had no effect on organs weight such as brain, heart, spleen, trachea, testis ventral prostrate and uterus as compared to the control group. The test drug administered at three different dose levels has shown significant reduction in the lung, liver and significant increase in the stomach and seminal vesicle weight as compared to normal control.

**Table 2:** Effect of different dose levels of GR on organs weight of Wistar albino rats.

Organs weight (g)	Groups							
	Control		GRTEDx½		GR TED		GR 5X TED	
	Male	Female	Male	Female	Male	Female	Male	Female
Brain	1.68 ± 0.03	1.7 ± 0.07	1.64 ± 0.09	1.59 ± 0.04	1.60 ± 0.03	1.59 ± 0.03	1.65 ± 0.05	1.59 ± 0.03
Lungs	3.55 ± 0.59	1.95 ± 0.18	2.28 ± 0.06∗	1.64 ± 0.15	1.93 ± 0.18∗∗	1.38 ± 0.05∗∗	2.03 ± 0.17∗	1.67 ± 0.04
Liver	9.66 ± 0.36	9.27 ± 0.36	10.55 ± 0.67	7.80 ± 0.34∗	8.31 ± 0.33	5.86 ± 0.34∗∗	11.75 ± 0.99	7.65 ± 0.35∗
Kidney	1.96 ± 0.07	1.55 ± 0.05	1.93 ± 0.06	1.31 ± 0.07	2.25 ± 0.07	1.20 ± 0.03∗∗	2.04 ± 0.12	1.40 ± 0.10
Heart	0.93 ± 0.01	0.79 ± 0.03	0.97 ± 0.04	0.71 ± 0.10	1.00 ± 0.02	0.65 ± 0.02	0.94 ± 0.03	0.74 ± 0.04
Spleen	1.02 ± 0.08	0.65 ± 0.05	1.06 ± 0.17	0.72 ± 0.09	0.87 ± 0.09	0.56 ± 0.04	1.36 ± 0.16	0.71 ± 0.01
Trachea	0.30 ± 0.00	0.22 ± 0.04	0.44 ± 0.07	0.26 ± 0.03	0.28 ± 0.04	0.20 ± 0.02	0.37 ± 0.05	0.33 ± 0.03
Stomach	1.66 ± 0.03	1.59 ± 0.04	2.16 ± 0.07∗∗	1.54 ± 0.07	1.59 ± 0.05	1.35 ± 0.04∗	1.96 ± 0.12∗	1.68 ± 0.05
Jejunum	0.63 ± 0.08	0.83 ± 0.06	0.76 ± 0.06	0.67 ± 0.06	0.60 ± 0.02	0.56 ± 0.03∗∗	0.85 ± 0.09	0.59 ± 0.04∗
Testis	2.97 ± 0.06	–	3.15 ± 0.19	–	2.87 ± 0.12	–	2.99 ± 0.11	–
Seminal vesicle	0.64 ± 0.02	–	1.34 ± 0.04∗∗	–	0.98 ± 0.03∗∗	–	1.08 ± 0.08∗∗	–
Ventral Prostate	0.61 ± 0.07	–	0.83 ± 0.07	–	0.67 ± 0.08	–	0.57 ± 0.05	–
Ovary	–	0.70 ± 0.05	–	0.45 ± 0.04∗	–	0.41 ± 0.04∗∗	–	0.56 ± 0.10
Uterus		0.92 ± 0.10		1.02 ± 0.16		0.87 ± 0.05		1.02 ± 0.09

Data: Mean ± SEM, ∗p < 0.05, ∗∗p < 0.01 in comparison to control group. GR- *Gandhaka rasayana*.

### 3.3 Effect of GR on haematological parameters

Total WBC count was found to be moderately but significantly elevated in female rats receiving half TED dose (p < 0.01), while no significant change was observed at the higher dose levels as compared to the control group. GR did not show any effect on total platelet count at all the three dose level as compared to the control group. Inconsistent changes were observed in RBC related parameters. Haemoglobin level and PCV% was found to show marginal decreased at 5X TED dose level in female rats while no significant effect was observed at the other two dose level in both male and female rats. RBC count was significantly increased in male rats belonging to TED dose group while no significant effect was observed in both male and female rats of other two drug administered groups. MCV was marginally decreased in TED group and it was statistically significant in comparison to control group. MCH was significantly decreased in male rats of TED group and was significantly increased in female rats of TED and half TED groups in comparison to the control group. Remaining groups showed no significant change. Marginal but statistically significant decrease in MCHC was observed in female rats of half TED group while no significant effect was observed in both male and female rats of other groups in comparison to the control group. RDWCV% was found marginally elevated in female rats of half TED groups which were statistically significant in comparison to the control group rats. Marginal but statistically significant decrease was found in RDWSD in male rats of TED group as compared to the control group. In male and female rats of other groups, no significant effect was observed in both the parameters (Table 3). Repeated administration of GR administered at five times of therapeutic dose caused significant reduction in the haemoglobin, PCV and MCHC level in female rats as compared to the control group, whereas GR at half of the therapeutic dose has shown significant reduction in the MCHC, RDWCV, MCH and significant increase in the total WBC count as compared to the control group (p < 0.01). GR administered at therapeutic dose caused significant increase in the RBC, RDWSD level and significant reduction in the parameters such as MCV, MCHC and MCH level as compared to the control group.

**Table 3:** Effect of different dose levels of GR on haematological parameters in albino rats.

Parameters	Groups							
	Control		GR (TED)		GR (5X TED)		GR (TEDx½)	
	Male	Female	Male	Female	Male	Female	Male	Female
Haemoglobin g/dL	15.24 ± 0.21	15.6 ± 0.3	15.94 ± 0.33	15 ± 0.1	15.08 ± 0.27	14.26 ± 0.2∗∗	15.38 ± 0.41	14.96 ± 0.1
Total WBC count/mm^3^	10,000 ± 1780.2	9185.71 ± 798.64	11271.42 ± 1204.9	8225 ± 612.30	13,050 ± 897.68	9933.33 ± 1108.1	12,500 ± 1300.8	13,180 ± 1421.4∗
RBC count 106/μL	8.05 ± 0.16	7.71 ± 0.13	8.80 ± 0.22∗	7.81 ± 0.09	7.78 ± 0.18	7.33 ± 0.11	8.40 ± 0.29	7.87 ± 0.11
PCV (%)	43.31 ± 0.68	43.11 ± 0.83	44.54 ± 0.74	41.73 ± 0.32	42.80 ± 0.55	40.33 ± 0.48∗∗	43.4 ± 1.23	42.86 ± 0.40
MCV (fl/cell)	53.9 ± 0.53	55.94 ± 0.24	50.22 ± 0.57∗∗	53.5 ± 0.60	55.13 ± 0.89	55.08 ± 0.66	51.7 ± 0.37	54.56 ± 0.89
MCH (pg/cell)	18.85 ± 0.18	20.12 ± 0.1	17.9 ± 0.25∗	19.15 ± 0.2∗∗	19.38 ± 0.23	19.4 ± 0.2	18.26 ± 0.16	18.96 ± 0.2∗∗
MCHC (g/dL)	35.02 ± 0.1	36.14 ± 0.1	35.71 ± 0.2	35.88 ± 0.2	35.18 ± 0.2	35.33 ± 0.1	35.38 ± 0.1	34.86 ± 0.1∗∗
RDWCV (%)	14.38 ± 0.3	12.6 ± 0.1	14.1 ± 0.2	13.3 ± 0.2	14.2 ± 0.4	13.08 ± 0.2	14.76 ± 0.3	14.1 ± 0.8∗
RDWSD (%)	27.14 ± 0.3	24.97 ± 0.6	24.72 ± 0.5∗∗	24.52 ± 0.5	27.8 ± 0.5	27.2 ± 1.5	26.56 ± 0.4	25.55 ± 0.3
Platelet count (10^3^/ml)	6.89 ± 0.34	6.95 ± 0.48	7.21 ± 0.26	8.28 ± 0.28	6.71 ± 0.30	6.97 ± 0.44	7.53 ± 0.49	7.40 ± 0.44

Data: Mean ± SEM, ∗p < 0.05, ∗∗p < 0.01 in comparison to control group.

### 3.4 Effect of GR on biochemical parameters

The blood sugar level was found to be significantly elevated in male rats of half TED administered group in comparison to the control group male rats. However, no significant change could be observed in both male and female rats at higher dose levels. Serum cholesterol level was significantly elevated in female rats of half TED group and male rats of TED groups. No significant changes were observed at other dose levels. Serum triglyceride level was significantly decreased in male rats of 5X TED dose groups whereas no significant change was observed in other groups in comparison to the control group. SGOT activity was significantly decreased in male rats of TED dose group in comparison to the male rats of the control group. No significant change in this parameter was observed in the other two drug administered groups. SGPT was found to be significantly elevated in male rats of half TED and 5X TED dose groups; however, marginal non-significant decrease was observed in TED dose group in comparison to the male rats of control group. Surprisingly, a significant decrease was observed in female rats of TED dose group while no significant effect was observed in the two remaining test drug administered groups in comparison to the control group female rats. Moderate, but statistically significant decrease in serum alkaline activity was observed in both male and female rats of TED dose group while no significant effect was observed in other two test drug administered groups. Moderate, but statistically significant decrease in the serum total protein was observed in female rats of half TED group and male rats of TED dose group in comparison to the corresponding rats in control group. No significant change was observed in rats of other groups and sex. Moderate, but statistically significant increase was observed in serum albumin level in male rats in half TED group and female rats in TED dose group. Significant decrease in the serum albumin and creatinine was observed in female rats of half TED group. No significant effect was observed in the rats of other test drug dosed groups. No significant effect could be observed in both total and direct bilirubin level in test drug administered groups in comparison to the control group.

Serum urea level was not affected to significant extent in both male and female rats of half TED and TED dose administered groups. However, female rats in 5X TED exhibited moderate but statistically significant elevation but no such effect was observed in male rats. Significant decrease was observed in serum creatinine level in female rats of half TED and TED dose administered groups. However, no significant effect was observed in female rats in 5X TED dose group and male rats in all the test drug administered groups.

Careful analysis shows that none of the studied parameters were significantly affected at all the three dose levels of GR. Observation of dose-dependent response in both sexes is considered to be the index of drug-related changes except for hormonal status dependent parameters. In that case, there may be difference in the responses observed between the sexes (Table 4). Repeated administration of GR at therapeutic dose has showed significant increase in the serum cholesterol and albumin level as compared to the control group, whereas the serum SGOT, SGPT, ALP and total bilirubin level was significantly decreased as compared to the control group. GR administered at five times of therapeutic dose has shown significant increase in the serum SGPT level whereas triglyceride level was significantly decrease as compared to the control group rats. GR administered at half the therapeutic dose has shown significant increase in the serum sugar, cholesterol, SGPT and albumin level and significant decrease in the serum creatinine and albumin level as compared to control group.

**Table 4:** Effect of different dose levels of GR *on* biochemical parameters in albino rats.

Parameters	Groups							
	Control		GR (TED)		GR (5X TED)		GR (TEDx½)	
	Male	Female	Male	Female	Male	Female	Male	Female
Blood sugar mg/dL	105.85 ± 7.2	128.85 ± 7.63	116.28 ± 8.85	113.25 ± 9.56	133.33 ± 3.85	143.33 ± 5.3	161.2 ± 12.71∗∗	108.8 ± 5.72
Cholesterol mg/dL	47.14 ± 2.25	53 ± 2.28	47.28 ± 2.60	80.5 ± 5.28∗∗	41.50 ± 3.24	63.6 ± 9.59	67.20 ± 3.33∗∗	49.16 ± 3.28
Triglycerides mg/dL	88.85 ± 9.34	122.42 ± 16.50	67.57 ± 4.02	100.37 ± 17.71	56.83 ± 10.62∗	87.16 ± 8.60	65.80 ± 9.25	87 ± 16.55
SGOT IU/L	139.28 ± 6.22	109.7 ± 13.0	96.71 ± 12.39∗∗	95.3 ± 5.56	164.16 ± 10.11	128 ± 8.04	134.60 ± 7.54	138.2 ± 14.14
SGPT IU/L	63.71 ± 1.89	60.85 ± 4.51	50.42 ± 5.84	35.5 ± 6.91∗	88.5 ± 6.34∗	66.33 ± 8.36	92.8 ± 8.42∗∗	69.2 ± 5.93
ALP IU/L	486.14 ± 89.70	489.7 ± 64.24	200.14 ± 32.35∗	244 ± 48.07∗	652.83 ± 83.86	584.6 ± 105	629.2 ± 92.03	288.4 ± 81.28
total protein g/dL	7.04 ± 0.11	7.05 ± 0.08	5.15 ± 0.35∗∗	7.61 ± 0.09	7.53 ± 0.22	7.01 ± 0.08	6.76 ± 0.12	5.94 ± 0.59∗
Albumin g/dL	3.51 ± 0.16	3.91 ± 0.09	3.02 ± 0.138	4.52 ± 0.08∗	3.75 ± 0.13	3.88 ± 0.05	4.10 ± 0.07∗	3.28 ± 0.34∗
Total bilirubin mg/dL	0.16 ± 0.02	0.08 ± 0.01	0.08 ± 0.00	0.16 ± 0.02	0.12 ± 0.01	0.10 ± 0.02	0.14 ± 0.01	0.30 ± 0.20
Direct bilirubin mg/dL	0.08 ± 0.02	0.07 ± 0.01	0.04 ± 0.01	0.09 ± 0.01	0.04 ± 0.00	0.03 ± 0.01	0.04 ± 0.00	0.07 ± 0.01
Urea mg/dL	34.71 ± 1.10	30.28 ± 1.58	29.85 ± 2.53	37.12 ± 1.66	29.83 ± 0.87	42.83 ± 2.97∗∗	34.0 ± 2.82	29 ± 4.95
Creatinine mg/dL	0.37 ± 0.01	0.55 ± 0.04	0.35 ± 0.02	0.32 ± 0.03∗∗	0.21 ± 0.03	0.44 ± 0.03	0.40 ± 0.13	0.19 ± 0.02∗∗

Data: Mean ± SEM, ∗p < 0.05, ∗∗p < 0.01 in comparison to control group.

### 3.5 Histopathology

**Fig. 1:**
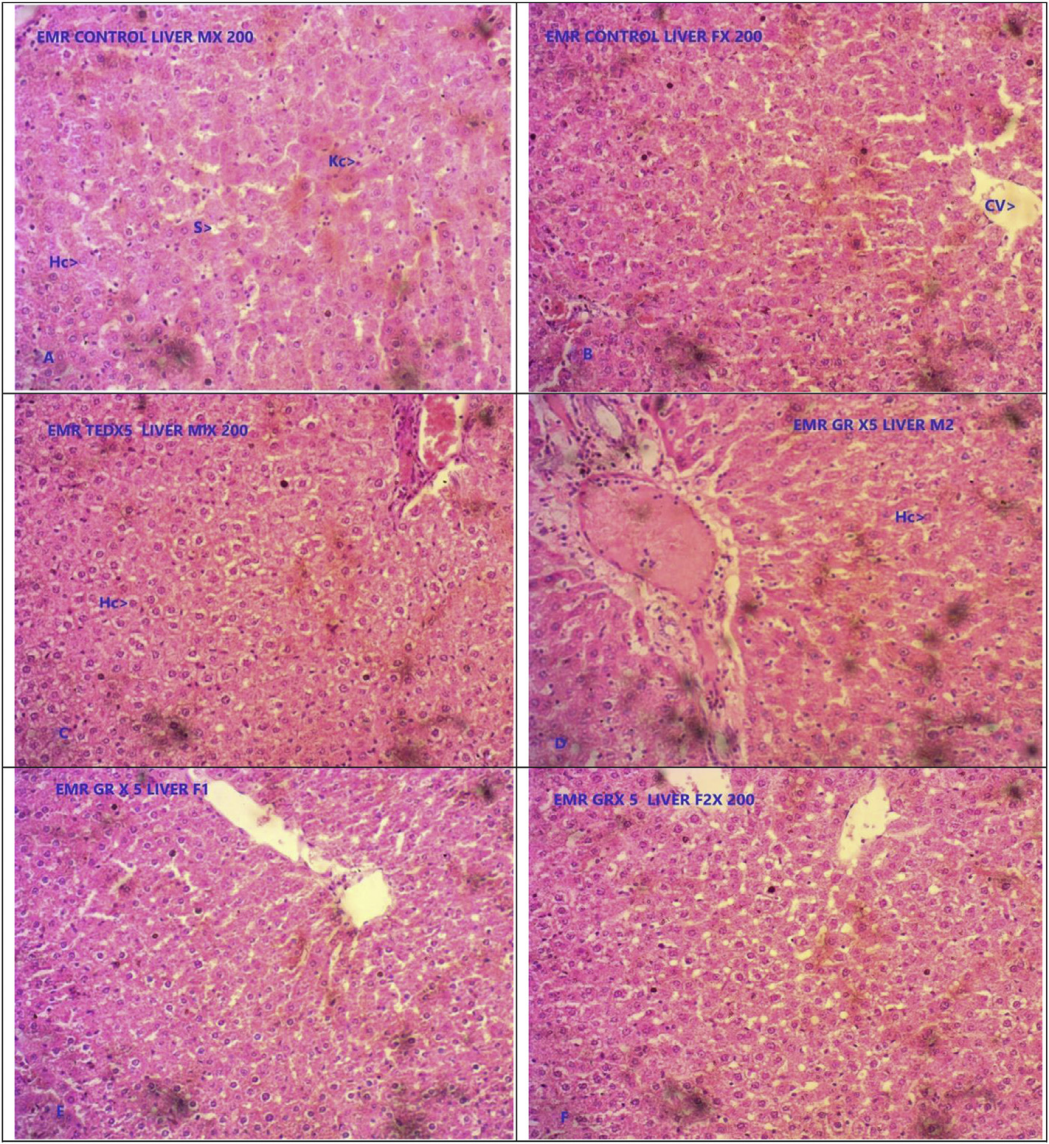
Photomicrograph of representative sections of liver tissues A & B- Control group C, D, E & F GR administered at five times of therapeutic dose (5X TED) male and female rats. Kc- Kuffer’s cells, Hc- Hepatic cell, HG- Hemorrhages, FC- Fatty changes, SD- Sinusoidal dilatation.

**Fig. 2:**
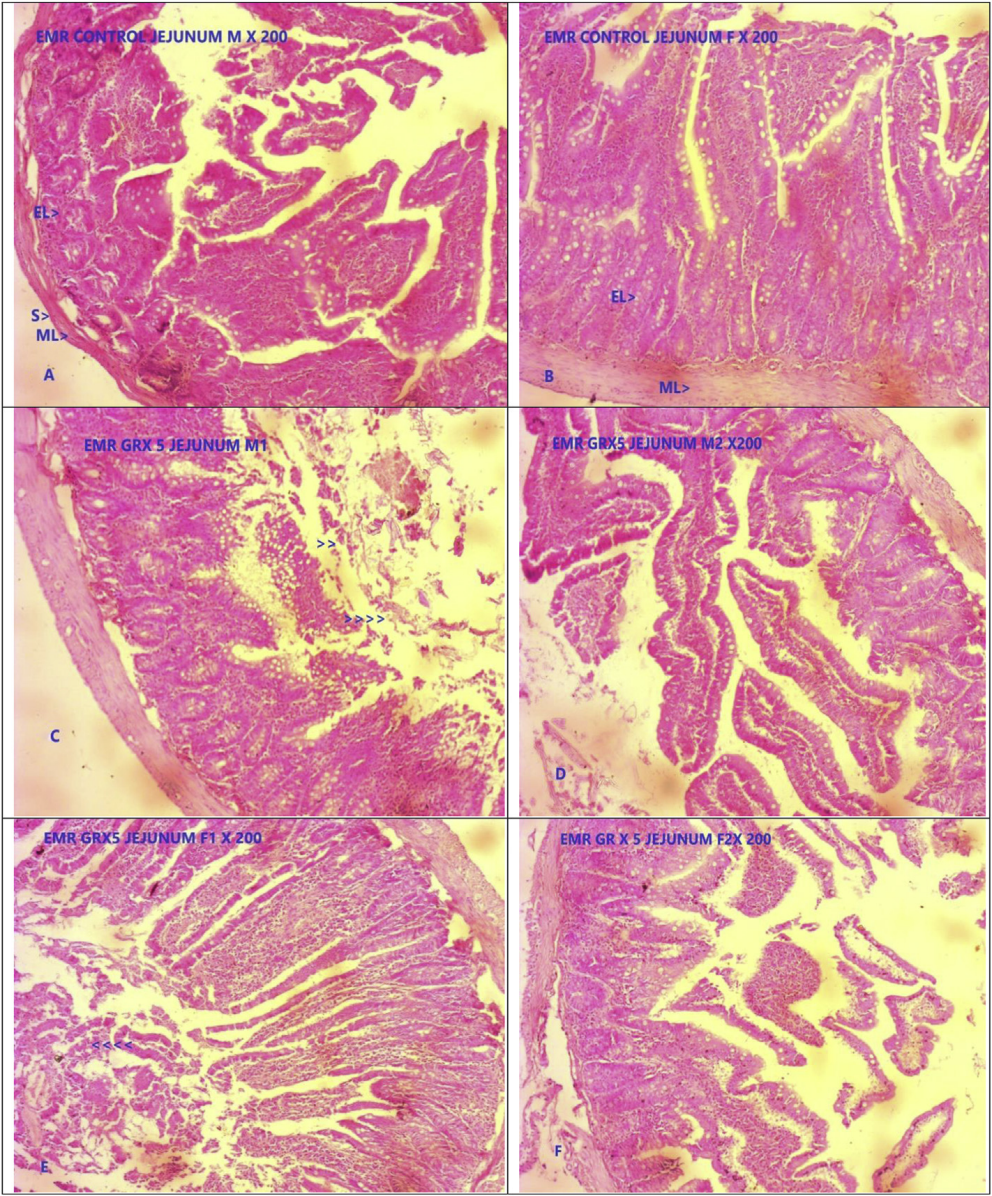
Photomicrograph of representative sections of Jejunum tissues A & B- Control group, C, D, E, F GR administered at five times of therapeutic dose (5X TED) male and female rats. SM- Smooth muscle, EL- Epithelial layer, ML- Muscle layer.

**Fig. 3:**
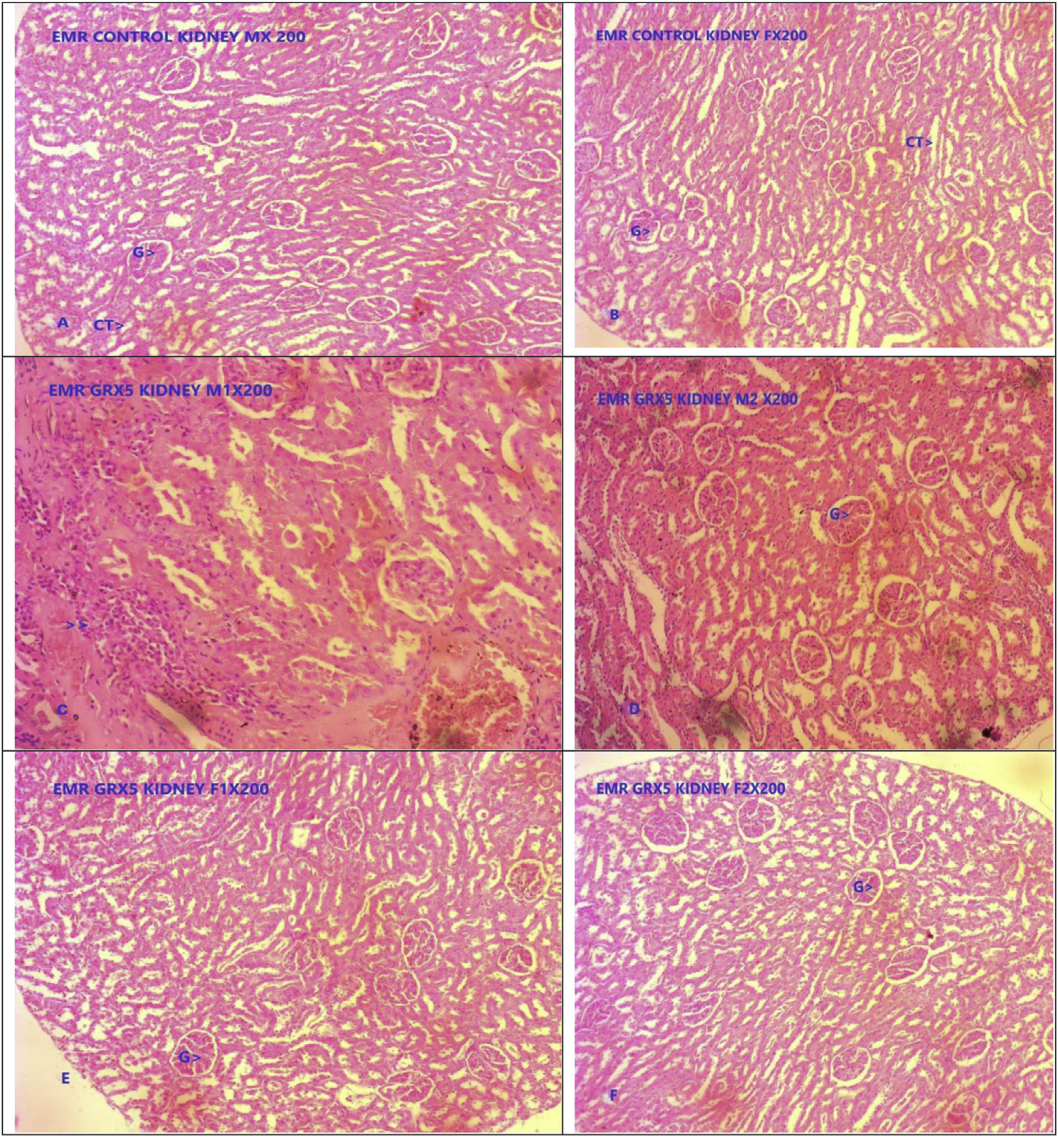
Photomicrograph of representative sections of kidney tissues A & B- Control group, C, D, E & F GR administered at five times of therapeutic dose (5X TED) male and female rats. G- Glomerulus, CI- Cell infiltration, CT- Central triad.

**Fig. 4:**
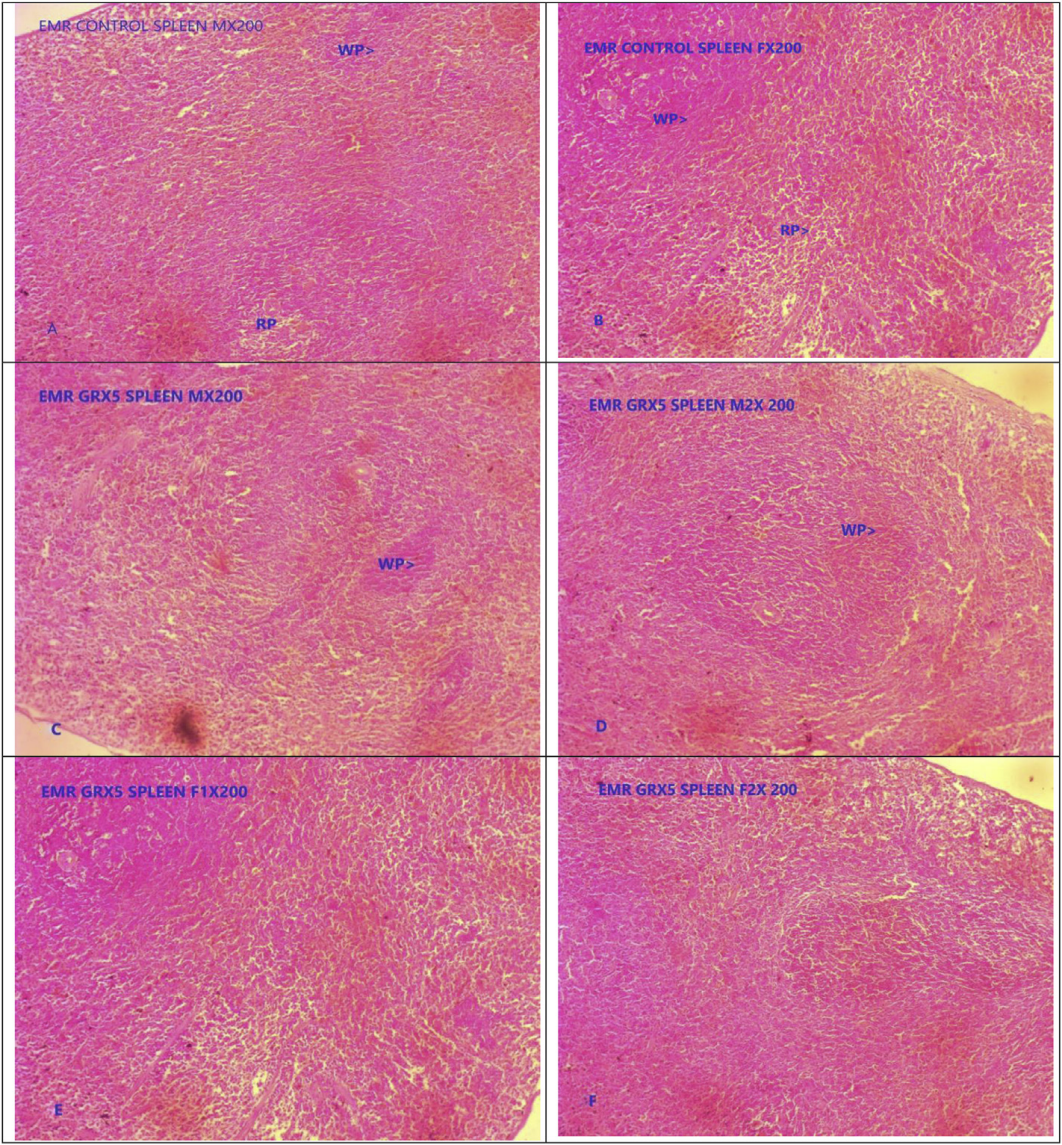
Photomicrograph of representative sections of spleen tissues A & B- Control group, C, D, E & F GR administered at five times of therapeutic dose (5X TED) male and female rats. WP- White pulp, RP- Red pulp, Cp- Capsule.

Repeated dose administration of GR didn't produce any noticeable histopathological changes in the organs such as brain, heart, lymph node, trachea, colon, uterus, ovary, seminal vesicles, and adrenal glands. However, the group in which GR was administered at five times the therapeutic dose, a mild haemorrhage was observed in kidney sections from one rat. All other rats were observed to have a normal cytoarchitecture. Histopathology of liver showed mild fatty changes in liver sections from two rats, and mild cellular infiltration in liver section from one rat. There was an increase in white pulp proportion of spleen in two female and three male rats in test drug administered group. The histological examination of jejunum showed mild epithelial erosion, and shortening of epithelial layer in one rat in GR administered at five times of therapeutic dose (Fig. 1, Fig. 2, Fig. 3, Fig. 4).

## 4 Discussion

GR is an important *Rasayana* drug used in Ayurveda. It has widespread clinical use in immune and skin related diseases. However, this indiscriminate clinical use lacks essential studies on its chronic toxicity profile. Hence, the effect of GR was studied to elucidate repeated dose toxicity profile. The 180 days study was carried out to evaluate the possible health hazards likely to arise from repeated exposure to the test formulation over a prolonged period of time [13]. Hence, the current study focused on analysing information related to major toxic effects on target organs for their toxic effects, and possibility of accumulation. The results can also be used to predict whether the test formulation has the potential to cause neurotoxic, immunological or reproductive organ-related adverse effects. An in *vitro* study by Pramod et al, reported that *gandhakadi* activates and modulates the expression of proteins involved in tissue remodelling. Further, cytoprotective effect activity has been reported with a *Gandhaka* preparation against iron overload-induced organ toxicity [2]. *Kajjali* is used as a base for numerous preparations. Safety evaluation of *Kajjali* *per se* revealed it to be quite safe in rats. Though GR has not been evaluated experimentally for safety aspects, many formulations containing *Gandhaka* derivatives, especially *kajjali*, have been studied and proven to be safe. It is also used in the form of *Kajjali*, a combination of mercury and sulphur in a number of preparations. An acute and sub-acute toxicity study on *Kajjali* powder suspension has no toxicity. Review of toxicological aspects of *Kajjali* revealed that several *Kajjali* prepared formulations are safe [17]. Several studies with *Kajjali* or other form of sulphur containing formulations also showed that they are safe [[12], [18]]. Extensive review of the published literature has shown that many herbomineral and metallic preparations including those based on *Kajjali* have proven safety and efficacy. Further, an *in vitro* lethality test has also showed that it is well-tolerated. Our present study also shows that GR does not produce significant toxicity even at doses which are not likely to be used clinically.

In the repeated dose toxicity study GR was found to be well-tolerated even at the highest dose level. It is interesting to note that the body weight gain was significant at all the dose levels throughout the study period in comparison to the control indicating the *rasayana* (rejuvenator) nature of the formulations. Body weight gain change is an important index of degenerative effect. The data obtained shows that GR even at the highest dose and for long administration (26 weeks) has no potential to cause any serious or significant degenerative changes or loss of tissue. On the contrary, tendency for the increase in body weight was observed, providing evidence for the *rasayana* nature of the drug.

Analysis of the haematological parameters indicates almost normal profile at three dose levels as compared to the control group. Changes in PCV, MCH, MCHC, RDW-SD level were marginally higher than those of normal control and observed changes were not dose-dependent. Thus, it can be considered that they do not indicate any pathological change [19].

Analysis of the biochemical parameters was done to ascertain if any dose-dependent remarkable changes have occurred in both sexes. No change could be observed meeting these criteria. However, statistically significant moderate changes were observed in many parameters. The implication of these changes was carefully analyzed. Serum cholesterol was found to be elevated in female rats of TED dose group and male rats of half TED dose group. SGPT was found to be moderately elevated in male rats of half TED and since these effects were not found at higher dose and in both the sexes it was inferred that it has no pathological significance. Further, SGPT elevation was attended with elevation of SGOT normally and both get elevated when there were degenerative changes. In addition, histopathological examination of liver, heart, and other organs did not reveal any significant degenerative changes. The apparent but statistically significant decrease observed in SGOT level in male rats of TED was inferred to have no toxicological significance in the context of the explanation given above.

Significant changes in serum protein and serum albumin were found in some groups. Total proteins get elevated when there is enzyme induction or there is remarkable change in the nitrogenous material turnover. Moderate but statistically significant increase in total protein was observed in only male rats in half TED and TED dose groups in contrast to this serum albumin level in male rats in half TED and female rats in TED group however, female of half TED group exhibited decrease. Since the changes were not dose-dependent, not seen in both groups and for high dose level, they were considered to have no pathological implications.

As the representative bio-markers of kidney function, the serum urea and the creatinine levels were recorded. In xenobiotic-induced injury, both the parameters get elevated. In the present study, however, the serum urea was moderately elevated only in female rats at 5X TED dose level and serum creatinine level was found to be decreased in female rats of half TED and TED dosed groups. Thus, there was no dose-dependent elevation in these biomarkers and increase seen in serum urea was in the highest dose given group. Such doses are not likely to be used in clinical settings. Also, considering that creatinine was lowered only in some rats, it can be unequivocally inferred that they do not indicate any impairment in renal function [20].

Analysis of the data related to organ weight showed increase or decrease in some of the organs; however, the changes were not consistent or dose-dependent. Histopathology did not reveal any significant degenerative changes in those organs except for epithelial erosion in jejunum and mild fatty changes in liver of some rats thus, they were considered to be of no pathological implication.

## 5 Conclusion

The data generated during the present study clearly showed that GR is safer for clinical use at the prescribed dose level. However, at high dose level (5.4 g/kg) which is five times higher than therapeutic dose and for a very long period of administration for rats, chances of mild organ toxicity can be observed. No ponderal, haematological, and clinical chemistry parameters were present at all the three dose levels and in both the sexes which is the requirement to consider any change as pathologically significant. Hence, the current study suggests that the test formulation is safe for human administration.

## Scope of future work

Further studies are required to assess the test drug for its mutagenicity and genotoxicity potential on repeated dosing.

## Source(s) of funding

This work is a part of the extramural research funded by Ministry of AYUSH, Govt. of India to Ravi Mundugaru (Z.28,015/209/2015-HPC-EMR). We acknowledge the intramural funds and laboratory facilities extended by SDM Centre for Research in Ayurveda and Allied Sciences, Udupi, India.

## Conflict of interest

None.
